# Metabolomic analysis of plasma and brain tissues in fentanyl-induced conditioned place preference mice

**DOI:** 10.3389/fphar.2026.1759491

**Published:** 2026-02-10

**Authors:** Kaili Du, Xiaoyu Ning, Yongze Han, Xiaoyu Jiang, Zhuoyi Wang, Tao Wang, Hongliang Su, Ting Liu, Bin Cong, Caixia Cheng, Keming Yun

**Affiliations:** 1 Department of Pathology, School of Basic Medicine, Shanxi Medical University, Taiyuan, China; 2 School of Forensic Medicine, Shanxi Medical University, Taiyuan, China; 3 Children’s Hospital of Shanxi Province, Taiyuan, China; 4 Zhaidian Police Station of Jishan County Public Security Bureau, Yuncheng, China; 5 Taizhou Municipal People’s Procuratorate, Taizhou, China; 6 School of Forensic Medicine, Hebei Medical University, Shijiazhuang, China; 7 Department of Pathology, the First Clinical Medical College, Shanxi Medical University, Taiyuan, China

**Keywords:** caudate putamen, conditioned place preference, fentanyl, hippocampus, metabolomics, plasma, prefrontal cortex

## Abstract

Fentanyl abuse has been associated with neurological and psychological harm. However, the metabolic changes within the peripheral circulation and central nervous system involved in fentanyl addiction have not been well explored. In the present study, metabolic changes in plasma, caudate putamen (CPu), hippocampus (Hip), and prefrontal cortex (PFC) were investigated in mouse models of drug addiction based on fentanyl-induced conditioned place preference (CPP). Metabolic profiles were measured using untargeted UHPLC-Q-Exactive HFX mass spectrometry. A total of 131, 196, 104, and 52 altered metabolites were identified in plasma, CPu, Hip, and PFC, respectively. The identified metabolites mainly included lipid mediators, carbohydrate metabolites, fatty acids, amino acids (AAs) and their derivatives, and nucleotide metabolites. The disturbed metabolic pathways were primarily involved in lipid metabolism, carbohydrate metabolism, AA metabolism, nucleotide metabolism, and the metabolism of cofactors and vitamins. These findings indicate disturbances in cell membrane metabolism, energy metabolism, AA metabolism, and neurotransmitter systems caused by fentanyl addiction. Our study provides a valuable resource for future investigations aimed at defining the role of metabolites in fentanyl addiction, which may help develop new pharmacotherapies.

## 1 Introduction

Drug abuse is a major global public health and social challenge that adversely affects physical and mental health and contributes to substantial socioeconomic burdens (Strang et al., 2020). The 2023 World Drug Report by the United Nations Office on Drugs and Crime (United Nations Office on Drugs and Crime (UNODC), 2023) estimated that in 2021, over 296 million people worldwide had used a drug in the past 12 months, of whom 60 million people engaged in non-medical opioid use, accounting for 1.2% of the global population. The opioid crisis in North America continues unabated, mainly attributed to the unprecedented high number of deaths caused by the use of fentanyl. Long-term drug abuse can seriously damage the structure and function of the brain, leading to compulsive nonmedical drug seeking and use despite negative health and social effects, which is so-called drug addiction (Wise and Robble, 2020). Fentanyl is a synthetic opioid that was initially manufactured for anesthesia and severe pain management; in addition to its clinical use, it is a powerfully addictive drug and its illegal use is related to the addiction crisis in the United States (Volkow, 2021). Research suggests that fentanyl abuse causes persistent changes in the physical structure and physiology of the brain, thus producing addictive behaviors that are difficult to reverse (Han et al., 2022).

Drug addiction has been proposed to involve profound metabolic dysregulation that contributes to persistent neurochemical and behavioral alterations (Caspani et al., 2022; Ghanbari and Sumner, 2018; Okeoma et al., 2025; Xu et al., 2021). By providing comprehensive biochemical data from biological samples, metabolomics aims to capture the metabolic state that reflects the overall health status of an individual (Quinones and Kaddurah-Daouk, 2009). To achieve different research objectives, metabolomics can be conducted through targeted and untargeted methods; the former aims to identify a limited number of specific metabolites and perform absolute quantification, while the latter focuses on acquiring as many known and unknown metabolites as possible and performing relative quantification (Schrimpe-Rutledge et al., 2016). Currently, the four major analytical platforms used in metabolomics include liquid chromatography-mass spectrometry (LC-MS), gas chromatography-mass spectrometry (GC-MS), nuclear magnetic resonance (NMR) spectroscopy, and capillary electrophoresis-mass spectrometry (CE-MS). Among these platforms, ultra-high-performance liquid chromatography coupled to high-resolution mass spectrometry (UHPLC-HRMS) is widely used in untargeted metabolomics research due to its high sensitivity, high peak resolution, and broad metabolome coverage across physicochemical properties and concentration ranges (Perez de Souza et al., 2021).

Given that chronic opioid exposure induces persistent neurochemical and metabolic adaptations in the brain, metabolomics offers a powerful systems-level approach to characterize these alterations (Zaitsu et al., 2016). In recent years, metabolomics has been applied to investigate the biochemical basis of the addiction cycle and predict prognosis by understanding the biochemical disorders associated with substance abuse (Caspani et al., 2022). Previous investigations have reported unique metabolic alterations in opioid addiction and have primarily focused on morphine and heroin abuse (Dinis-Oliveira, 2019). Regarding fentanyl, studies have shown metabolic alterations in fentanyl-overdose mouse models using urine, liver, and nucleus accumbens (NAc) samples (Alasmari et al., 2023; Alasmari et al., 2024; Di Francesco et al., 2024). The NAc (located in the ventral striatum) is a major neural node of the mesolimbic dopaminergic system (MLDS) which guides animals to addictive drugs. Dopamine (DA) neurons in mesolimbic regions such as the ventral tegmental area (VTA) and substantia nigra (SN) send projections to the NAc, caudate putamen (CPu, located in the dorsal striatum), amygdala (Amy), prefrontal cortex (PFC), and hippocampus (Hip), regions traditionally associated with action selection, reinforcement learning, and encoding and retrieval of conditioning to drug cues (Volkow et al., 2017). However, these studies primarily focused on overdose or toxicity models and did not address reward-related learning or metabolic alterations across interconnected mesolimbic brain regions. Additionally, profiling peripheral blood metabolites may provide clinically accessible biomarkers that reflect central nervous system alterations associated with fentanyl dependence. Therefore, metabolomic studies of the blood and associated brain regions underlying fentanyl dependence are promising for uncovering the pharmacological mechanisms of addiction therapy.

Researchers have developed a number of animal models, including conditioned place preference (CPP), intracranial self-stimulation (ICSS), drug self-administration (SA), and so on, to investigate the specific pathological brain processes underlying addiction. Compared to other animal models, CPP reflects associative learning between drug reward and environmental context, a process critically involving the Hip and PFC. The CPP paradigm provides a measure of motivated behaviors and is widely used to explore the mechanisms underlying the rewarding properties of addictive drugs (Garcia Pardo et al., 2017; Spanagel, 2017). Therefore, the CPP paradigm in mice was used as an animal model in our present study to explore the mechanism of fentanyl-induced rewarding behavior. The metabolites in the plasma, CPu, PFC, and Hip of fentanyl-dependent mice were profiled using UHPLC-HRMS, following a robust untargeted metabolomics workflow. This study aimed to characterize fentanyl-induced metabolic reprogramming in mesolimbic brain regions and plasma in order to define the global metabolic signature of the integrated fentanyl-CPP phenotype, thereby elucidate underlying metabolic pathways and reveal potential peripheral biomarkers of fentanyl dependence.

## 2 Materials and methods

### 2.1 Animals

Sixteen male C57BL/6 wild-type mice (aged 6 weeks and weighing 18–22 g upon arrival at the laboratory) were obtained from the Research Animal Center of Shanxi Medical University. Sample size was determined based on previous CPP and metabolomics studies and feasibility considerations. Only male mice were used to minimize variability related to estrous cycle–associated hormonal fluctuations. The mice were housed in pairs in a temperature-controlled room (22 °C ± 2 °C) under a 12 h light/dark cycle. Humidity levels were maintained at 45% ± 10%, and food and water were available *ad libitum*. They were habituated through daily handling for 1 week prior to experiments. Mice were randomly assigned to experimental groups, and behavioral analysis was performed by an investigator blinded to treatment. All experimental procedures involving animals were performed in strict accordance with the ARRIVE guidelines and under a protocol approved by the Institutional Animal Care and Use Committee (IACUC) of Shanxi Medical University.

### 2.2 Chemicals and reagents

Fentanyl citrate injection (0.05 mg/mL) was provided by Yichang Humanwell Pharmaceutical Co., Ltd. (Hubei, PR China) and was diluted with sterile saline to the relevant concentration on the day of use. LC-MS-grade ammonium acetate and ammonium hydroxide were purchased from Sigma-Aldrich (United States) and Fisher Chemical (United States), respectively. LC-MS-grade methanol and acetonitrile were acquired from CNW Technologies (Shanghai, PR China). Ultra-pure water was obtained using a Milli-Q system (Millipore, Bedford, United States).

### 2.3 CPP procedure

The CPP apparatus (JLBeHv, PR China) consisted of two identically sized conditioning compartments (15 cm × 15 cm × 37 cm) separated by a sliding door (15 cm × 37 cm). The compartments differed in wall color, light intensity, and floor texture to provide distinct visual and tactile cues. One black compartment had white walls, dim lighting, and a metal grid floor, while the other white compartment had black walls, bright lighting, and a metal rod floor. The black compartment was illuminated at approximately 30 lux, and the white compartment at 15 lux (measured at the center of each compartment). This differential was consistently maintained throughout all phases of the experiment. An infrared monitoring system within the apparatus tracked mouse position and movement, recording compartment crossings and time spent in each compartment.

**FIGURE 1:**
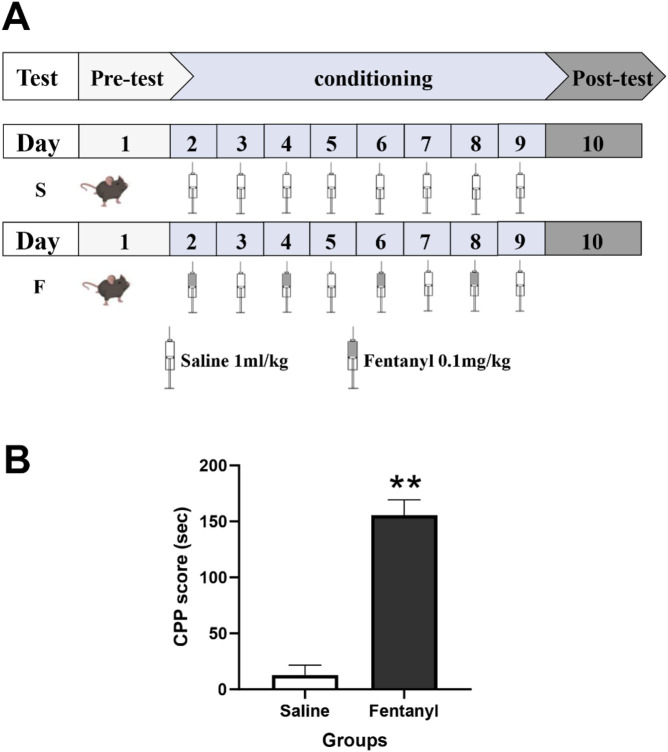
The procedure and results of conditioned place preference (CPP). **(A)** The CPP procedure consisted of three phases: pre-test phase (Day 1, drug-free), conditioning phase (Days 2–9; drug training), and post-test phase (Day 10, drug-free). S: mice in the control group (saline), F: mice in the fentanyl group. **(B)** Effect of fentanyl on place conditioning (mean ± S.E.M., n = 8 per group). **P < 0.01 compared with the saline group.

The CPP procedure consisted of three phases: pre-test (Day 1, drug-free), conditioning (Days 2–9; drug training), and post-test (Day 10, drug-free) (Figure 1A). An unbiased CPP procedure was used. The pre-test phase determined baseline preference. All mice were drug-free and randomly placed in the central area with the sliding door removed to explore both compartments freely for 15 min. The time each mouse spent in each compartment and the number of crossings between compartments were recorded. Any mouse that spent >600 s in either compartment or crossed <20 times was considered to have compartment bias and was excluded immediately following the completion of the pre-test phase, prior to the commencement of any conditioning sessions. No animals were replaced after exclusion. The final cohort for both the fentanyl and saline groups consisted of n = 8 mice each. During conditioning, mice were divided into a fentanyl group and a control group. Fentanyl was paired with the less preferred compartment (white) during conditioning phase. Each mouse received its corresponding treatment at the same time daily. On Day 2, the fentanyl group received an intraperitoneal (i.p.) injection of fentanyl (0.1 mg/kg) and the control group received saline; both groups were immediately confined to the white compartment for 40 min. The dosage of fentanyl employed in this study was derived from our prior research, which established its efficacy in eliciting robust CPP and aligns closely with both clinical administration ranges and recreational usage patterns observed in humans (Du et al., 2021; Comer and Cahill, 2019; Hughes et al., 2023). The i.p. injection volume was 10 mL/kg. On Day 3, all mice received saline and were confined to the black compartment for 40 min. Each conditioning session comprised two consecutive days of training. For the fentanyl group, mice received a fentanyl injection before being confined to the white compartment on the first day, and a saline injection before confinement to the black compartment on the second day. Control mice received saline injections before confinement to both compartments on both days. All mice underwent four conditioning sessions (Days 2–9). During the post-test phase (Day 10), all drug-free mice explored both compartments freely for 15 min under pre-test conditions. Residence times in each compartment were recorded. All behavioral experiments were performed during the light phase (8:00 a.m.–6:00 p.m.). Behavioral data acquisition and analysis were conducted by investigators blinded to treatment groups.

### 2.4 Sample preparation

On Day 10, immediately after completing the post-test, blood collection was performed under anesthesia to minimize animal distress. Blood samples were collected from the orbital venous plexus using heparinized Eppendorf (EP) tubes. Plasma was separated by centrifugation at 4,000 rpm for 15 min at 4 °C, snap-frozen in liquid nitrogen, and stored at −80 °C until analysis. After blood collection, mice were euthanized by cervical dislocation following deep anesthesia, and death was confirmed prior to tissue collection. Whole brains were rapidly removed, and brain regions were dissected according to standard mouse brain atlases cite reference, snap-frozen in liquid nitrogen, and stored at −80 °C.

For metabolite extraction, 100 µL of plasma was mixed with 400 μL of pre-cooled extraction solvent stored at −20 °C (acetonitrile/methanol, 1:1 v/v) and vortexed for 30 s. The mixture was sonicated for 10 min in an ice-water bath, incubated for 1 h at −40 °C, and then centrifuged (12,000 rpm, 15 min, 4 °C) to precipitate proteins. The supernatant was transferred to glass LC vials for analysis. Brain regions (10 mg each) were homogenized with 200 μL of pre-cooled extraction solvent stored at −20 °C (acetonitrile/methanol/water, 2:2:1 v/v/v) in a ball mill at 35 Hz for 4 min, followed by sonication for 5 min in an ice-water bath. After repeating this homogenization-sonication cycle three times, samples were incubated for 1 h at −40 °C and centrifuged at 12,000 rpm for 15 min at 4 °C. The resulting supernatants were transferred to glass LC vials for UHPLC-HRMS analysis. Quality control (QC) samples were prepared by pooling equal aliquots of each sample’s supernatant to monitor system and analytical method stability.

### 2.5 UHPLC-HRMS analysis

Samples were analyzed using a UHPLC system (Vanquish, Thermo Fisher Scientific, United States) coupled to a Q Exactive HFX mass spectrometer (Orbitrap MS, Thermo Fisher Scientific, United States). Metabolites were separated on an Acquity UPLC BEH Amide column (2.1 mm × 100 mm, 1.7 μm; Waters Associates, United States), the column temperature was maintained at 30 °C. The mobile phase consisted of water containing 25 mmol/L ammonium acetate and ammonium hydroxide (pH 9.75) (A) and acetonitrile (B). The gradient elution program was as follows: 0–0.5 min, 95% B; 0.5–7 min, 95% → 65% B; 7–8 min, 65% → 40% B; 8–9 min, 40% B; 9–9.1 min, 40% → 95% B; 9.1–12 min, 95% B. Untargeted data were collected over a mass range of m/z 70–1,200 in both positive and negative ionization modes. The auto-sampler temperature was maintained at 4 °C, and the flow rate was 0.3 mL/min. The injection volume was 2 μL.

Under control of the acquisition software (Xcalibur, Thermo), the Q Exactive HFX mass spectrometer acquired full-scan MS spectra in information-dependent acquisition (IDA) mode. The instrument was operated with electrospray ionization (ESI) in positive (ESI+) and negative (ESI-) ion modes. ESI source parameters were: sheath gas flow rate 30 arb, aux gas flow rate 25 arb, full MS resolution 60,000, MS/MS resolution 7,500, spray voltage 3.6 kV (positive) or −3.2 kV (negative), collision energy 10/30/60 in stepped NCE mode, and capillary temperature 350 °C.

### 2.6 Data processing and metabolite identification

The raw data files were converted to mzXML format using ProteoWizard. Peak detection, extraction, alignment, and integration were subsequently processed using the R package XCMS. The key parameters for XCMS were set as follows: the mass accuracy for chromatographic peak detection was 10 ppm; the peak width range was 5–20 s; and the snthresh was set to 3. Features detected in <50% of QC samples or with high coefficient of variation (CV > 30%) were filtered. The dataset was then normalized by sum normalization, log-transformed, and autoscaled before being subjected to multivariate statistical analysis using SIMCA software (Umetrics, Umeå, Sweden, ver. 14.1). Multivariate analyses including principal component analysis (PCA) and orthogonal partial least squares discriminant analysis (OPLS-DA) were performed for pattern recognition. PCA, an unsupervised dimensionality reduction method, transforms original variables into principal components (PCs), whereby the resulting score plots thus enable the visualization of sample similarity and diversity. OPLS-DA, a supervised method, determines inter-group variation. Seven-fold cross-validation (using R2 and Q2 parameters) evaluated model quality, and 200 response permutation tests (RPT) prevented overfitting. Significantly changed metabolites were screened using variable importance in projection (VIP) >1 from OPLS-DA and *P* < 0.05 from Student’s t-test. Metabolite annotation utilized an in-house MS/MS database and public databases (KEGG, METLIN, HMDB), and was assigned a level 2 identification based on the Chemical Analysis Working Group of the Metabolomics Standards Initiative (Viant et al., 2017). Metabolic pathway analysis was performed using the Metabolomic Pathway Analysis (MetPA) module within the MetaboAnalyst platform.

### 2.7 Statistical analysis

The CPP score was calculated as the difference in residence time between the pre-test and post-test phases for each mouse in the drug-paired compartment. SPSS 22.0 software was used for statistical analysis. All data are presented as mean ± SEM. Student’s t-test was performed to analyze fentanyl’s effects on place conditioning. A *P* -value <0.05 was considered statistically significant.

## 3 Results

### 3.1 Fentanyl-induced CPP

The majority of mice showed a slight, non-statistically significant tendency toward the black compartment during pre-testing (mice spent 52% ± 4% of time in the black compartment and 48% ± 4% in the white compartment; *P* > 0.05), indicating a natural preference for darkness. Therefore, we proceeded with an unbiased design and systematically paired fentanyl with the white compartment. As shown in Figure 1B, significant CPP developed after four fentanyl conditioning sessions (**P < 0.01 vs. saline group). Fentanyl-treated mice showed a mean CPP score of 155.6 ± 13.7 s, whereas saline-treated mice exhibited no compartment preference (12.77 ± 8.7 s). The locomotor activity (total distance traveled) of both the fentanyl and saline groups during the post-test session did not differ significantly from their own baseline levels, nor was there a significant difference between the two treatment groups (*P* > 0.05) (post-test: 3026 ± 112 cm vs. 2998 ± 93 cm; baseline: 3032 ± 98 cm vs. 3055 ± 115 cm).

### 3.2 Multivariate statistical analysis

**FIGURE 2:**
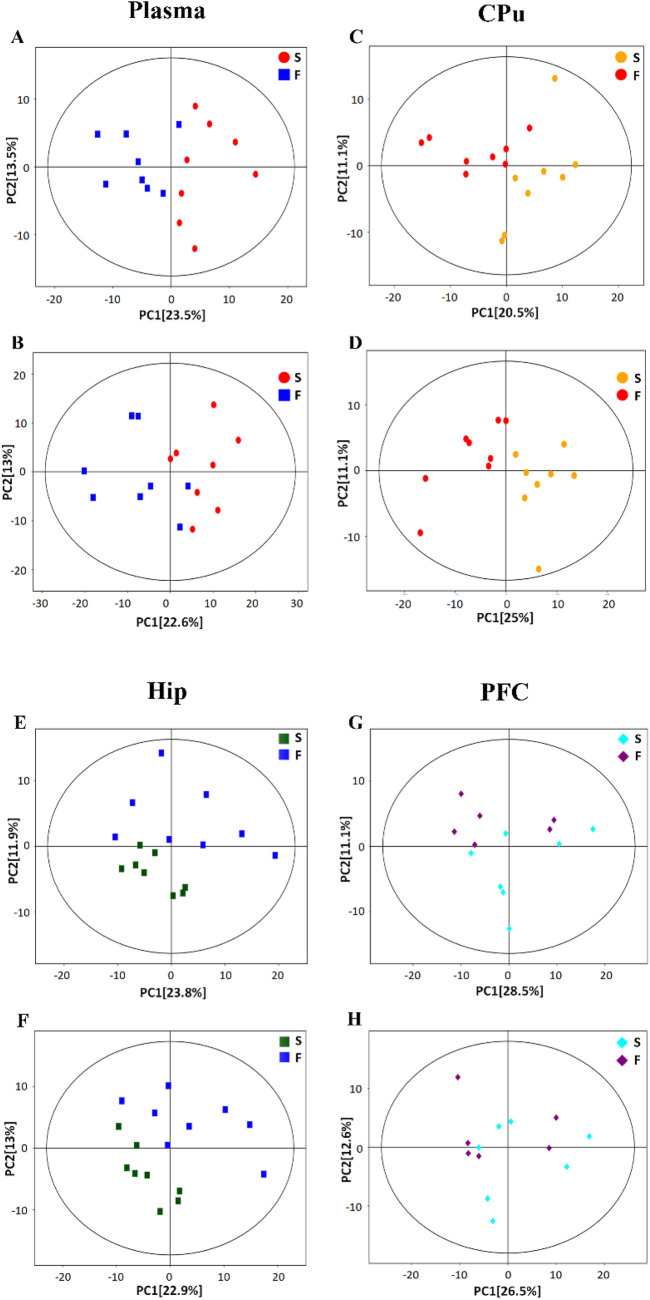
The PCA score plots of plasma, CPu, Hip, and PFC samples from the control group and fentanyl group. PCA reduces the dimensionality of the dataset by transforming numerous detected variables into a few derived variables called principal components (PCs). PC1 is the first principal component of the model, and PC2 is the second. The percentage in parentheses represents the variance explained by each PC. The PCA score plots show the scatter of samples; closely clustered samples indicate similar metabolome compositions, while those farther apart indicate different compositions. Ellipses display 95% confidence regions. **(A,C,E,G)** Show data collected in positive ionization mode. **(B,D,F,H)** Show data collected in negative ionization mode. S: mice in the control group (saline), F: mice in the fentanyl group.

**FIGURE 3:**
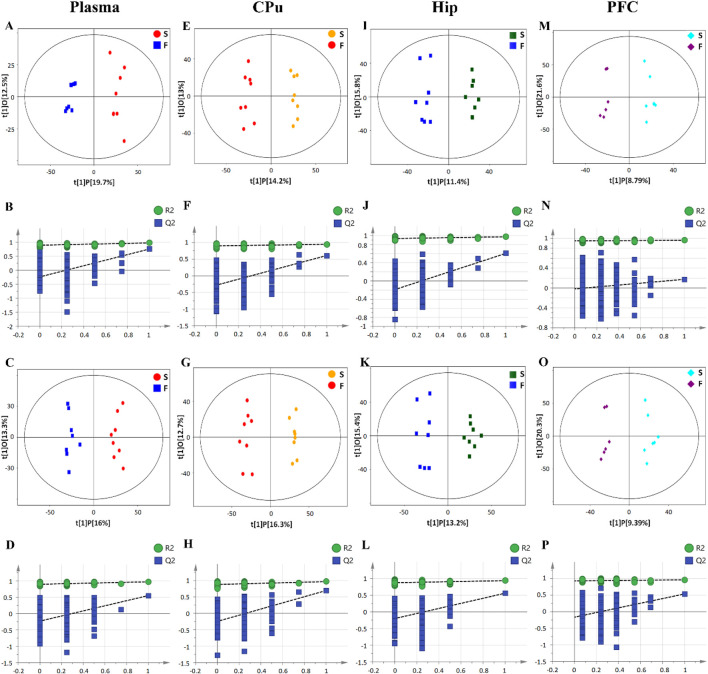
OPLS-DA score plots of plasma **(A,C)**, CPu **(E,G)**, Hip **(I,K)**, and PFC **(M,O)** samples from the control and fentanyl groups. OPLS-DA was utilized to maximize differentiation between groups and identify metabolites contributing to separation. P1 and O1 represent the first predictive component and first orthogonal component of the model, respectively. The percentage in parentheses represents the variance explained by the component. Ellipses display 95% confidence regions. Validation plots from 200 permutation tests for OPLS-DA models of plasma **(B,D)**, CPu **(F,H)**, Hip **(J,L)**, and PFC **(N,P)**. Lower R2 and Q2 values for random permutation models indicate good reliability of the original OPLS-DA model. A negative Q2 intercept indicates good model predictability. **(A,B,E,F,I,J,M,N)** Data collected in positive ionization mode. **(C,D,G,H,K,L,O,P)** Data collected in negative ionization mode. S: mice in the control group (saline), F: mice in the fentanyl group.

PCA was first performed to visualize sample clustering and identify potential. In Figure 2, the first two principal components (PCs) showed samples scattered in distinct regions, suggesting distinct metabolic profiles associated with fentanyl treatment in plasma, CPu, Hip, and PFC. All samples remained within the 95% confidence Hotelling’s T2 ellipse. An OPLS-DA model was subsequently established to maximize differentiation between fentanyl and control groups and identify separation-contributing metabolites. Model reliability was confirmed through 200 response permutation tests (RPTs) (Figure 3). Cross-validation yielded R2Y and Q2 values demonstrating good model interpretability and predictability. In validation plots, permuted Y-vector R2 and Q2 values were lower than the original models, confirming OPLS-DA reliability. Negative Q2 intercepts indicated good model fit and predictive ability. Model parameters are shown in Supplementary Table S1.

### 3.3 Metabolomic alterations in fentanyl-CPP mice

Based on screening criteria, differentially altered metabolites in plasma, CPu, Hip, and PFC were identified in both positive and negative ionization modes (Supplementary Table S2). For the plasma, CPu, Hip, and PFC, a total of 131 (85 in the positive mode, 49 in the negative mode), 196 (134 in the positive mode, 71 in the negative mode), 104 (68 in the positive mode, 37 in the negative mode), and 52 (31 in the positive mode, 23 in the negative mode) metabolites were changed in fentanyl-CPP mice, respectively. These metabolites primarily included lipid mediators, carbohydrate metabolites, fatty acids, amino acids (AAs) and their derivatives, and nucleotide metabolites. To streamline interpretation of the complex metabolomics data, Supplementary Table S3 presents significantly altered metabolites, organized by their associated metabolic pathways.

#### 3.3.1 Lipid metabolism

Phospholipids, including glycerophospholipids and sphingomyelin, are major structural components of biological membranes. In our study, glycerophospholipids such as phosphatidylcholines (PC), phosphatidylserines (PS), phosphatidylethanolamines (PE), and phosphatidylinositols (PI) were increased in CPu, Hip, PFC, and plasma of the fentanyl group. Myo-inositol (M-Ins), a significant intracellular osmolyte which is considered a marker of astrocytic activity, was increased in plasma, while its derivative D-myo-inositol 4-phosphate (belonging to inositol phosphates) was increased in CPu and Hip.

#### 3.3.2 Carbohydrate metabolism

Glucose, a primary energy substance for brain metabolism, was increased in plasma and the three brain regions in the fentanyl group. Correspondingly, the intermediate products of glucose metabolism increased: 3-phosphoglyceric acid was increased in the three brain regions; dihydroxyacetone phosphate was increased in CPu and Hip; glyceric acid and itaconic acid were increased in plasma and CPu. Creatinine, the cyclic form of the well-known high energy compound creatine, was also increased in plasma, CPu, and PFC. In addition, ketone bodies, which serve as energy sources alternative to glucose, were decreased in the fentanyl group: acetoacetic acid (AcAc) was decreased in plasma, and 3-hydroxybutyric acid (3-HB) was decreased in CPu and PFC.

#### 3.3.3 Amino acid metabolism

Tyrosine was increased in plasma and the three brain regions in the fentanyl group. Valine, arginine, proline, leucine, isoleucine, tryptophan, phenylalanine, glycine, serine, lysine, citrulline, and argininosuccinic acid were upregulated in CPu. Cystine, serine, and homocysteic acid were upregulated in Hip while lysine, ornithine, and citrulline were upregulated in plasma. Methionine was upregulated in PFC. As metabolites of histidine, urocanic acid and formiminoglutamic acid were increased in PFC, ergothioneine was increased in CPu, and 3-methylhistidine was decreased in CPu and Hip in the fentanyl group. Significantly, derived from enzymatic decarboxylation of histidine, the monoamine neurotransmitter histamine was only increased in plasma while its metabolite imidazoleacetic acid was increased in plasma, CPu, and Hip. Metabolites of histidine, including 5-hydroxy-L-tryptophan and indoleacetaldehyde, were decreased in plasma in fentanyl-CPP mice.

#### 3.3.4 Nucleotide metabolism

The levels of uridine as well as its biosynthetic intermediates and catabolites changed significantly in the fentanyl group. Uridine *de novo* synthesis originates from glutamine (Gln) and produces the intermediate metabolites carbamoyl phosphate, carbamoyl aspartate, dihydroorotate, and orotidine. The feedstock Gln was increased in plasma and CPu while uridine was decreased in CPu. Among intermediate metabolites, dihydroorotate levels increased in CPu, while orotidine levels increased in CPu, Hip, and plasma. Among uridine catabolites, uracil was decreased in CPu, while β-alanine was decreased in plasma and Hip. Additionally, the feedstock of purine nucleotides, including Gln, aspartate, serine, and glycine, were all increased but the intermediate metabolites adenosine and deoxyguanosine were decreased in CPu. Meanwhile, the catabolites of purine nucleotides, including xanthine, uric acid and guanine, were increased in CPu, Hip, and both, respectively. As to nucleoside derivatives, N6-methyladenosine (m6A) decreased in plasma yet increased in the CPu, whereas N4-acetylcytidine (ac4C) decreased in both CPu and Hip.

#### 3.3.5 Vitamin/cofactor metabolism

Acting as cofactors for enzymes that play a vital role in converting carbohydrates and fats into energy, B vitamins showed significant alterations in the fentanyl group. Vitamin B1 (thiamine) increased in CPu; its active form thiamin pyrophosphate (TPP) serves as a coenzyme for α-ketoacid oxidative dehydrogenase complexes. Nicotinamide adenine dinucleotide (NAD^+^), the active form of vitamin B3, decreased in CPu. Pyridoxal (vitamin B6 family) increased in CPu and Hip. Metabolites of vitamins B3, B5 (pantothenic acid), B6, and B7 (biotin)—including nicotinamide mononucleotide (NMN), β-alanine, uracil, 4-pyridoxic acid, and lysine—underwent significant changes in plasma and relevant brain regions. Additionally, vitamin C (ascorbic acid) increased in PFC.

### 3.4 Alterations in metabolic pathways

**FIGURE 4:**
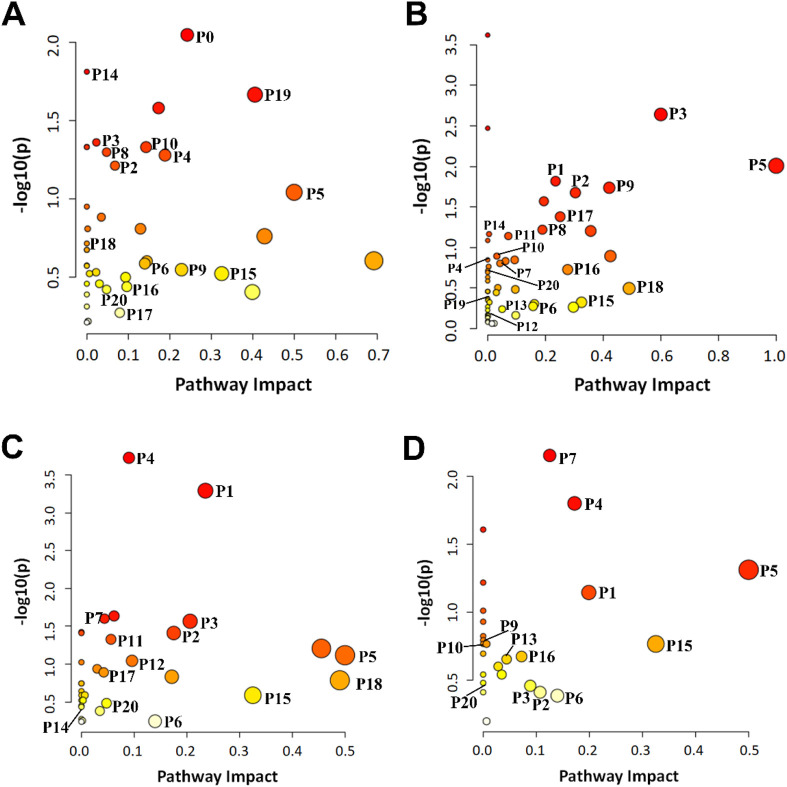
Metabolic pathways of differential metabolites identified in plasma **(A)**, CPu **(B)**, Hip **(C)**, and PFC **(D)**. Each circle represents a metabolic pathway. The color intensity (shade) of each circle corresponds to the pathway enrichment *P*-value (darker red indicates greater significance), while the circle size represents the pathway impact value (larger circles indicate greater impact). P1: glycerophospholipid metabolism; P2: pyrimidine metabolism; P3: glycine, serine and threonine metabolism; P4: histidine metabolism; P5: phenylalanine, tyrosine and tryptophan biosynthesis; P6: tyrosine metabolism; P7: cysteine and methionine metabolism; P8: arginine and proline metabolism; P9: arginine biosynthesis; P10: butanoate metabolism; P11: purine metabolism; P12: glycolysis/gluconeogenesis; P13: citrate cycle (TCA cycle); P14: pentose phosphate pathway; P15: starch and sucrose metabolism; P16: pentose and glucuronate interconversions; P17: glyoxylate and dicarboxylate metabolism; P18: vitamin B6 metabolism; P19: ascorbate and aldarate metabolism; P20: pantothenate and CoA biosynthesis.

Metabolomic pathway analysis (MetPA) revealed that fentanyl-induced CPP broadly dysregulated fundamental metabolic processes, with alterations concentrated in lipid, carbohydrate, and amino acid metabolism (Figure 4). A core set of pathways was consistently disrupted across multiple brain regions and plasma, highlighting systemic metabolic reprogramming. These included glycerophospholipid metabolism, glyoxylate and dicarboxylate metabolism, glycine, serine and threonine metabolism, histidine metabolism, pyrimidine and purine metabolism. Notably, lipid metabolism pathways (e.g., glycerophospholipid, arachidonic acid) showed the most extensive alterations across tissues, while disturbances in the citrate cycle (TCA cycle) and glycolysis/gluconeogenesis were more region-specific (primarily in CPu and Hip). The enriched pathways are visualized in a bubble plot (Figure 4), with their complete listing and specific tissue distributions compiled in Supplementary Table S4.

## 4 Discussion

In the present study, fentanyl disrupted glycerophospholipid metabolism, increasing PC, PS, PE, and PI levels indicating membrane damage. Dysregulated M-Ins and D-myo-inositol 4-phosphate further support membrane disruption and suggest altered astrocytic activity, which affects neurotransmission and brain energy metabolism in addiction (Miguel-Hidalgo, 2009). Indeed, specific glycerophospholipids act as messengers, linking their dysregulation to cognitive disorders (Bou Khalil et al., 2010; Yu et al., 2020). Furthermore, altered lipid metabolism may compensate for heightened energy demands in addiction (Caspani et al., 2022).

Neuronal activity is extremely energy demanding, and the energy supply of the brain is almost entirely dependent on the oxidative metabolism of glucose in mitochondria. In fentanyl-CPP mice, we observed elevated glucose levels, suggestive of disrupted energy homeostasis and oxidative stress (Mei et al., 2013). This coincides with increased intermediates of glycolysis and the TCA cycle in key brain regions, collectively indicating an upregulation of carbohydrate metabolism to meet heightened energy requirements. Concurrently, the rise in Gln—which fuels the TCA cycle beyond its role as a glutamate (Glu) precursor—and its conversion to Glu further supports enhanced *de novo* synthesis and energy production (Brown and Yamamoto, 2003). In parallel, the elevation of creatinine across plasma and brain tissues (CPu, PFC) points to a protective response against energy depletion and oxidative damage. Conversely, levels of ketone bodies (AcAc, 3-HB, acetone), which serve as alternative brain fuels during glucose scarcity (Cotter et al., 2013; Morris, 2005), were decreased. This suppression of fatty acid β-oxidation and ketogenesis (Zaitsu et al., 2014) suggests a metabolic shift away from lipid-derived energy sources. Taken together, the coordinated elevation of glucose-related metabolites and reduction in ketone bodies reveal a metabolic reprogramming favoriting carbohydrates over lipids for energy supply in fentanyl dependence.

To date, purine and pyrimidine metabolites have been proven to be involved in various neuropsychiatric disorders, such as depression (Yao et al., 2013), neurodegenerative diseases (Burnstock, 2008), and drug addiction (Liu et al., 2017; Mannelli et al., 2009; Patkar et al., 2009). In the present study, purine and pyrimidine metabolism was significantly changed in the fentanyl group. Consistent with prior studies (Li et al., 2011; Liu et al., 2007; Song et al., 2013; Yang et al., 2006), the observed changes in nucleotide metabolites indicate that fentanyl administration inhibits the anabolism and enhances the catabolism of purine nucleotides in the brain, increasing uridine release from the CPu and Hip. The increased guanine, adenine and xanthine may indicate the conversion into their corresponding nucleotides through salvage pathways which could alter energy production. In fact, adenosine and uridine serve as potential endogenous neuromodulators in the CNS that might be responsible for modification of addictive behaviors through interact with the endocannabinoid (EC), γ-aminobutyric acid (GABA) receptors, serotoninergic, and dopaminergic system to regulate DA-related behaviors (Guarneri et al., 1985; Guarneri et al., 1983; Kimura et al., 2001; Pasquini et al., 2022). Attributed to the inhibitory effect on enhanced DA release and the ability to acquire associations between reward and cues, uridine, uracil, and β-alanine could also significantly attenuate opioid dependence and addictive behaviors (Liu et al., 2017; Liu et al., 2014; Song et al., 2013). In the same way, adenosine receptor agonists could significantly attenuate opioid dependence, while adenosine antagonists exacerbate these effects in animals (Kaplan and Sears, 1996; Listos et al., 2011; Wu et al., 2013). Notably, nucleoside derivatives M6A and ac4C—the most common RNA modifications involved in chromatin remodeling and transcriptional regulation—are implicated in neurogenesis, synaptic function, learning, memory, and reward (Broly et al., 2022; Shafik et al., 2022). Studies confirm that persistent OUD stems partly from long-term neuronal plasticity changes driven by transcriptomic (Huggett et al., 2022) and epigenetic (Browne et al., 2020) shifts. Our results further support that M6A and ac4C modifications regulate neural responses to opioids and may drive opioid-induced transcriptomic dysregulation (Dabrowski and Daws, 2024).

Elevated free AAs like valine, arginine, and proline likely reflect protein hydrolysis-induced negative nitrogen balance (Murin et al., 2009; Wu et al., 2009). Concomitantly, increased urea cycle intermediates (arginine, ornithine, citrulline, argininosuccinate) further confirm nitrogen imbalance. Notably, these AAs—alongside leucine, isoleucine, serine, and citrulline—modulate TCA cycle flux. Glycine and serine additionally support purine nucleotide synthesis, glycerophospholipid metabolism, creatine production, and pyruvate cycling. Collectively, these changes indicate dysregulated energy metabolism. Elevated arginine and methionine may counteract oxidative stress as reactiveoxygenspecies (ROS) scavengers (Liang et al., 2018; Luo and Levine, 2009). Furthermore, altered methionine and its precursor homocysteine imply disrupted transmethylation—critical for epigenetic regulation of DNA/RNA expression. Elevated histidine and histamine metabolites suggest enhanced catabolism of these compounds in the brain. Previous studies report significantly reduced histamine levels in the VTA and NAc of morphine-CPP rats—likely due to morphine-triggered histamine depletion from nerve terminals. Simultaneously, both histidine and histamine inhibit morphine-induced CPP by suppressing dopaminergic activity (Gong et al., 2007). Although we observed no direct decrease of histidine/histamine in reward-related brain regions, increased metabolites in these regions—along with peripheral histamine elevation—may indicate weakened inhibition of fentanyl’s rewarding effects.

Aromatic amino acids (AAs)—phenylalanine, tyrosine, and tryptophan—regulate key neurotransmitter pathways. Phenylalanine converts to tyrosine, a precursor of catecholamines (CAs, including tyramine, DA, epinephrine, norepinephrine/NE). Dysregulated phenylalanine/tyrosine metabolism associates not only with depression and neurodegeneration but also impairs DA transmission in the MLDS (Hufner et al., 2019; Volkow et al., 2017). Elevated plasma 3-Methoxy-4-hydroxyphenylethyleneglycol sulfate (an NE metabolite) further indicates CAs disturbances. Tryptophan generates serotonin (5-HT), whose deficiency contributes to opioid withdrawal symptoms (Welsch et al., 2020). Reduced plasma tryptophan metabolites suggest compensatory brain uptake to maintain 5-HT levels (Zaitsu et al., 2014). Collectively, these AAs abnormalities could disrupt catecholaminergic/monoaminergic neurotransmission—particularly for DA and 5-HT, which cannot cross the blood-brain barrier directly.

Compared to saline controls, fentanyl increased Gln in plasma and CPu. Paradoxically, GABA decreased in plasma but increased in CPu. These shifts likely reflect fentanyl-induced disruption of the Gln-Glu-GABA axis—a conversion pathway where Gln metabolizes to GABA via Glu. Elevated valine, arginine, and proline (which modulate Gln/Glu metabolism) further support this dysregulation (Meng et al., 2012). GABA is an endogenous GABA receptor agonist. Opioid reinforcement involves co-activation of GABA-A and µ receptors on GABAergic interneurons. This suppresses GABA release from VTA interneurons onto DA nerve endings, disinhibiting DA neurons and enhancing DA efflux (Saigusa et al., 2008). Based on our results, fentanyl likely mediates reinforcement through this GABAergic disinhibition pathway (Sustkova-Fiserova et al., 2017). In fact, Increased GABA in MLDS may represent an adaptive response to opioids (Hu et al., 2012). GABA also contributes to DA-independent opioid reinforcement via EC systems (Befort, 2015; Fields and Margolis, 2015; Maldonado et al., 2006; Vigano et al., 2005) 61–64. Cannabinoid CB1 receptor (CB1) and µ-receptors co-localize on GABA presynaptic terminals (Fields and Margolis, 2015; Maldonado et al., 2006). CB1 antagonists could reduce opioid rewarding effects, while agonists enhance them (Singh et al., 2004; Solinas et al., 2003; Solinas et al., 2005). ECs (notably anandamide/AEA and 2-arachidonoylglycerol/2-AG) act as retrograde messengers, modulating GABA release via presynaptic CB1 activation (Piomelli, 2003). Heroin, morphine, and fentanyl increase AEA in reward circuits (Caille et al., 2007; Sustkova-Fiserova et al., 2017; Vigano et al., 2004)—a mechanism confirmed by primate self-administration studies (Justinova et al., 2005). Our data show that fentanyl elevates AEA in CPu and Hip, further implicating ECs in opioid reward.

Our study shows that perturbed vitamins regulate energy production and neurotransmission: TPP facilitates mitochondrial oxidative decarboxylation (pyruvate, α-ketoglutarate, branched chain AAs) and supports acetylcholine synthesis; NAD+ provides protons for oxidative phosphorylation; the active form of vitamin B5 including coenzyme-A (CoA) and acyl carrier protein (ACP) are essential cofactors in pyruvate/α-ketoglutarate dehydrogenase complexes; biotin (B7) enables carboxylation reactions in gluconeogenesis and fatty acid oxidation; vitamin C, recognized as an antioxidant and anti-inflammatory agent involved in glutathione recycling, further functions as an electron donor for CAs and 5-HT biosynthesis (Depeint et al., 2006a; b; Zelfand, 2020). Disrupted vitamin metabolism—combined with glucose dysregulation—impairs acetyl-CoA production and downstream pathways (fatty acid/carbohydrate metabolism). While substance abuse alters vitamin absorption (el-Nakah et al., 1979; Nabipour et al., 2014), we observed elevated plasma/brain levels of neurotransmission-associated vitamins in fentanyl-CPP mice. This selective upregulation suggests compensatory modulation of central neurotransmission.

## 5 Conclusion and future perspectives

In summary, the exploration of metabolite profiles in the plasma, CPu, PFC, and Hip of fentanyl-CPP mice provides valuable insights into the complex interplay between neurochemistry and the rewarding effects of fentanyl administration. Our results revealed disturbances in key metabolites and metabolic pathways associated with cell membrane metabolism, energy metabolism, AA metabolism, and neurotransmitter regulation within the MLDS of fentanyl-CPP mice. This finding not only contributes to elucidating the mechanism of the conditioned state associated with fentanyl-induced rewarding effect but also holds promise for developing novel interventions for fentanyl dependence in future research and prevention strategies.

While our sample size (n = 8 per group) was sufficient to detect robust and significant metabolic alterations associated with fentanyl-induced CPP, it remains a limitation common to exploratory metabolomics. Future studies with larger sample sizes are needed to confirm these findings, investigate less abundant metabolites, and reduce the false-negative rate. Nevertheless, the strong statistical signals and validated multivariate models presented here provide a solid foundation for such subsequent work.

Additionally, considering that the preference for addictive drugs fluctuates with estradiol levels during the estrous cycle in females (Gaulden et al., 2021; Quigley et al., 2021), our study focused primarily on male mice. In future research, given the growing emphasis on sex differences in neuroscience, investigating sex-specific metabolomic variations in fentanyl-dependent mice will yield more comprehensive neurochemical insights into fentanyl addiction mechanisms.

## Data Availability

The original contributions presented in the study are included in the article/Supplementary Material, further inquiries can be directed to the corresponding authors.

## References

[B1] Alasmari F. Alasmari M. S. Assiri M. A. Alswayyed M. Rizwan Ahamad S. Alhumaydhi A. I. (2023). Liver metabolomics and inflammatory profiles in mouse model of fentanyl overdose treated with beta-lactams. Metabolites 13, 965. 10.3390/metabo13080965 37623908 PMC10456707

[B2] Alasmari M. S. Alasmari F. Alsharari S. D. Alasmari A. F. Ali N. Ahamad S. R. (2024). Neuroinflammation and neurometabolomic profiling in fentanyl overdose mouse model treated with novel beta-Lactam, MC-100093, and ceftriaxone. Toxics 12, 604. 10.3390/toxics12080604 39195706 PMC11360732

[B3] Befort K. (2015). Interactions of the opioid and cannabinoid systems in reward: insights from knockout studies. Front. Pharmacol. 6, 6. 10.3389/fphar.2015.00006 25698968 PMC4318341

[B4] Bou Khalil M. Hou W. Zhou H. Elisma F. Swayne L. A. Blanchard A. P. (2010). Lipidomics era: accomplishments and challenges. Mass Spectrom. Rev. 29, 877–929. 10.1002/mas.20294 20931646

[B5] Broly M. Polevoda B. V. Awayda K. M. Tong N. Lentini J. Besnard T. (2022). THUMPD1 bi-allelic variants cause loss of tRNA acetylation and a syndromic neurodevelopmental disorder. Am. J. Hum. Genet. 109, 587–600. 10.1016/j.ajhg.2022.02.001 35196516 PMC9069073

[B6] Brown J. M. Yamamoto B. K. (2003). Effects of amphetamines on mitochondrial function: role of free radicals and oxidative stress. Pharmacol. Ther. 99, 45–53. 10.1016/s0163-7258(03)00052-4 12804698

[B7] Browne C. J. Godino A. Salery M. Nestler E. J. (2020). Epigenetic mechanisms of opioid addiction. Biol. Psychiatry 87, 22–33. 10.1016/j.biopsych.2019.06.027 31477236 PMC6898774

[B8] Burnstock G. (2008). Purinergic signalling and disorders of the central nervous system. Nat. Rev. Drug Discov. 7, 575–590. 10.1038/nrd2605 18591979

[B9] Caille S. Alvarez-Jaimes L. Polis I. Stouffer D. G. Parsons L. H. (2007). Specific alterations of extracellular endocannabinoid levels in the nucleus accumbens by ethanol, heroin, and cocaine self-administration. J. Neurosci. 27, 3695–3702. 10.1523/JNEUROSCI.4403-06.2007 17409233 PMC6672416

[B10] Caspani G. Sebok V. Sultana N. Swann J. R. Bailey A. (2022). Metabolic phenotyping of opioid and psychostimulant addiction: a novel approach for biomarker discovery and biochemical understanding of the disorder. Br. J. Pharmacol. 179, 1578–1606. 10.1111/bph.15475 33817774

[B11] Comer S. D. Cahill C. M. (2019). Fentanyl: receptor pharmacology, abuse potential, and implications for treatment. Neurosci. Biobehav. Rev. 106, 49–57. 10.1016/j.neubiorev.2018.12.005 30528374 PMC7233332

[B12] Cotter D. G. Schugar R. C. Crawford P. A. (2013). Ketone body metabolism and cardiovascular disease. Am. J. Physiol. Heart Circ. Physiol. 304, H1060–H1076. 10.1152/ajpheart.00646.2012 23396451 PMC3625904

[B13] Dabrowski K. R. Daws S. E. (2024). Morphine-driven m6A epitranscriptomic neuroadaptations in primary cortical cultures. Mol. Neurobiol. 61, 10684–10704. 10.1007/s12035-024-04219-z 38780720 PMC11584444

[B14] Depeint F. Bruce W. R. Shangari N. Mehta R. O'Brien P. J. (2006a). Mitochondrial function and toxicity: role of B vitamins on the one-carbon transfer pathways. Chem. Biol. Interact. 163, 113–132. 10.1016/j.cbi.2006.05.010 16814759

[B15] Depeint F. Bruce W. R. Shangari N. Mehta R. O'Brien P. J. (2006b). Mitochondrial function and toxicity: role of the B vitamin family on mitochondrial energy metabolism. Chem. Biol. Interact. 163, 94–112. 10.1016/j.cbi.2006.04.014 16765926

[B16] Di Francesco G. Montesano C. Vincenti F. Bilel S. Corli G. Petrella G. (2024). Tackling new psychoactive substances through metabolomics: UHPLC-HRMS study on natural and synthetic opioids in male and female murine models. Sci. Rep. 14, 9432. 10.1038/s41598-024-60045-2 38658766 PMC11043364

[B17] Dinis-Oliveira R. J. (2019). Metabolism and metabolomics of opiates: a long way of forensic implications to unravel. J. Forensic Leg. Med. 61, 128–140. 10.1016/j.jflm.2018.12.005 30621882

[B19] Du K. Wang Z. Zhang H. Zhang Y. Su H. Wei Z. (2021). Levo-tetrahydropalmatine attenuates the acquisition of fentanyl-induced conditioned place preference and the changes in ERK and CREB phosphorylation expression in mice. Neurosci. Lett. 756, 135984. 10.1016/j.neulet.2021.135984 34029649

[B20] el-Nakah A. Frank O. Louria D. B. Quinones M. A. Baker H. (1979). A vitamin profile of heroin addiction. Am. J. Public Health 69, 1058–1060. 10.2105/ajph.69.10.1058 484761 PMC1619165

[B21] Fields H. L. Margolis E. B. (2015). Understanding opioid reward. Trends Neurosci. 38, 217–225. 10.1016/j.tins.2015.01.002 25637939 PMC4385443

[B22] Garcia Pardo M. P. Roger Sanchez C. De la Rubia Orti J. E. Aguilar Calpe M. A. (2017). Animal models of drug addiction. Adicciones 29, 278–292. 10.20882/adicciones.862 28170057

[B23] Gaulden A. D. Burson N. Sadik N. Ghosh I. Khan S. J. Brummelte S. (2021). Effects of fentanyl on acute locomotor activity, behavioral sensitization, and contextual reward in female and male rats. Drug Alcohol Depend. 229, 109101. 10.1016/j.drugalcdep.2021.109101 34628096 PMC8671359

[B24] Ghanbari R. Sumner S. (2018). Using metabolomics to investigate biomarkers of drug addiction. Trends Mol. Med. 24 (2), 197–205. 10.1016/j.molmed.2017.12.005 29397321

[B25] Gong Y. X. Lv M. Zhu Y. P. Zhu Y. Y. Wei E. Q. Shi H. (2007). Endogenous histamine inhibits the development of morphine-induced conditioned place preference. Acta Pharmacol. Sin. 28, 10–18. 10.1111/j.1745-7254.2007.00470.x 17184577

[B26] Guarneri P. Guarneri R. Mocciaro C. Piccoli F. (1983). Interaction of uridine with GABA binding sites in cerebellar membranes of the rat. Neurochem. Res. 8, 1537–1545. 10.1007/BF00964155 6324012

[B27] Guarneri P. Guarneri R. La Bella V. Piccoli F. (1985). Interaction between uridine and GABA-mediated inhibitory transmission: studies *in vivo* and *in vitro* . Epilepsia 26, 666–671. 10.1111/j.1528-1157.1985.tb05709.x 2866953

[B28] Han Y. Cao L. Yuan K. Shi J. Yan W. Lu L. (2022). Unique pharmacology, brain dysfunction, and therapeutic advancements for fentanyl misuse and abuse. Neurosci. Bull. 38, 1365–1382. 10.1007/s12264-022-00872-3 35570233 PMC9107910

[B29] Hu Z. Deng Y. Hu C. Deng P. Bu Q. Yan G. (2012). (1)H NMR-based metabonomic analysis of brain in rats of morphine dependence and withdrawal intervention. Behav. Brain Res. 231, 11–19. 10.1016/j.bbr.2012.02.026 22391120

[B30] Hufner K. Fuchs D. Blauth M. Sperner-Unterweger B. (2019). How acute and chronic physical disease may influence mental health - an analysis of neurotransmitter precursor amino acid levels. Psychoneuroendocrinology 106, 95–101. 10.1016/j.psyneuen.2019.03.028 30959235

[B31] Huggett S. B. Ikeda A. S. McGeary J. E. Kaun K. R. Palmer R. H. C. (2022). Opioid use disorder and alternative mRNA splicing in reward circuitry. Genes (Basel) 13, 1045. 10.3390/genes13061045 35741807 PMC9222793

[B32] Hughes L. M. Irwin M. G. Nestor C. C. (2023). Alternatives to remifentanil for the analgesic component of total intravenous anaesthesia: a narrative review. Anaesthesia 78 (5), 620–625. 10.1111/anae.15952 36562193

[B33] Justinova Z. Goldberg S. R. Heishman S. J. Tanda G. (2005). Self-administration of cannabinoids by experimental animals and human marijuana smokers. Pharmacol. Biochem. Behav. 81, 285–299. 10.1016/j.pbb.2005.01.026 15932767 PMC2679508

[B34] Kaplan G. B. Sears M. T. (1996). Adenosine receptor agonists attenuate and adenosine receptor antagonists exacerbate opiate withdrawal signs. Psychopharmacology (Berl) 123, 64–70. 10.1007/BF02246282 8741956

[B35] Kimura T. Ho I. K. Yamamoto I. (2001). Uridine receptor: discovery and its involvement in sleep mechanism. Sleep 24, 251–260. 10.1093/sleep/24.3.251 11322706

[B36] Li K. He H. T. Li H. M. Liu J. K. Fu H. Y. Hong M. (2011). Heroin affects purine nucleotides metabolism in rat brain. Neurochem. Int. 59, 1104–1108. 10.1016/j.neuint.2011.10.001 22019714

[B37] Liang M. Wang Z. Li H. Cai L. Pan J. He H. (2018). l-Arginine induces antioxidant response to prevent oxidative stress via stimulation of glutathione synthesis and activation of Nrf2 pathway. Food Chem. Toxicol. 115, 315–328. 10.1016/j.fct.2018.03.029 29577948

[B38] Listos J. Talarek S. Poleszak E. Wrobel A. Fidecka S. (2011). Attenuating effect of adenosine receptor agonists on the development of behavioral sensitization induced by sporadic treatment with morphine. Pharmacol. Biochem. Behav. 98, 356–361. 10.1016/j.pbb.2011.01.019 21295055

[B39] Liu C. Liu J. K. Kan M. J. Gao L. Fu H. Y. Zhou H. (2007). Morphine enhances purine nucleotide catabolism *in vivo* and *in vitro* . Acta Pharmacol. Sin. 28, 1105–1115. 10.1111/j.1745-7254.2007.00592.x 17640470

[B40] Liu P. Wu C. Song W. Yu L. Yang X. Xiang R. (2014). Uridine decreases morphine-induced behavioral sensitization by decreasing dorsal striatal dopamine release possibly via agonistic effects at GABAA receptors. Eur. Neuropsychopharmacol. 24, 1557–1566. 10.1016/j.euroneuro.2014.06.010 25088943

[B41] Liu P. Che X. Yu L. Yang X. An N. Song W. (2017). Uridine attenuates morphine-induced conditioned place preference and regulates glutamate/GABA levels in mPFC of mice. Pharmacol. Biochem. Behav. 163, 74–82. 10.1016/j.pbb.2017.10.004 29024680

[B42] Luo S. Levine R. L. (2009). Methionine in proteins defends against oxidative stress. FASEB J. 23, 464–472. 10.1096/fj.08-118414 18845767 PMC2630790

[B43] Maldonado R. Valverde O. Berrendero F. (2006). Involvement of the endocannabinoid system in drug addiction. Trends Neurosci. 29, 225–232. 10.1016/j.tins.2006.01.008 16483675

[B44] Mannelli P. Patkar A. Rozen S. Matson W. Krishnan R. Kaddurah-Daouk R. (2009). Opioid use affects antioxidant activity and purine metabolism: preliminary results. Hum. Psychopharmacol. 24, 666–675. 10.1002/hup.1068 19760630 PMC3183957

[B45] Mei B. Wang T. Wang Y. Xia Z. Irwin M. G. Wong G. T. (2013). High dose remifentanil increases myocardial oxidative stress and compromises remifentanil infarct-sparing effects in rats. Eur. J. Pharmacol. 718, 484–492. 10.1016/j.ejphar.2013.07.030 23954793

[B46] Meng J. Zhang X. Wu H. Bu J. Shi C. Deng C. (2012). Morphine-induced conditioned place preference in mice: metabolomic profiling of brain tissue to find “molecular switch” of drug abuse by gas chromatography/mass spectrometry. Anal. Chim. Acta 710, 125–130. 10.1016/j.aca.2011.09.033 22123121

[B47] Miguel-Hidalgo J. J. (2009). The role of glial cells in drug abuse. Curr. Drug Abuse Rev. 2, 72–82. 19630738

[B48] Morris A. A. (2005). Cerebral ketone body metabolism. J. Inherit. Metab. Dis. 28, 109–121. 10.1007/s10545-005-5518-0 15877199

[B49] Murin R. Mohammadi G. Leibfritz D. Hamprecht B. (2009). Glial metabolism of valine. Neurochem. Res. 34, 1195–1203. 10.1007/s11064-008-9895-2 19127430

[B50] Nabipour S. Ayu Said M. Hussain Habil M. (2014). Burden and nutritional deficiencies in opiate addiction- systematic review article. Iran. J. Public Health 43, 1022–1032. 25927032 PMC4411899

[B51] Okeoma C. M. Naushad W. Okeoma B. C. Gartner C. Santos-Ortega Y. Vary C. (2025). Lipidomic and proteomic insights from extracellular vesicles in the postmortem dorsolateral prefrontal cortex reveal substance use disorder-induced brain changes. Transl. Psychiatry 15, 284. 10.1038/s41398-025-03512-2 40817260 PMC12356847

[B52] Pasquini S. Contri C. Merighi S. Gessi S. Borea P. A. Varani K. (2022). Adenosine receptors in neuropsychiatric disorders: fine regulators of neurotransmission and potential therapeutic targets. Int. J. Mol. Sci. 23, 1219. 10.3390/ijms23031219 35163142 PMC8835915

[B53] Patkar A. A. Rozen S. Mannelli P. Matson W. Pae C. U. Krishnan K. R. (2009). Alterations in tryptophan and purine metabolism in cocaine addiction: a metabolomic study. Psychopharmacology (Berl) 206, 479–489. 10.1007/s00213-009-1625-1 19649617

[B54] Perez de Souza L. Alseekh S. Scossa F. Fernie A. R. (2021). Ultra-high-performance liquid chromatography high-resolution mass spectrometry variants for metabolomics research. Nat. Methods 18, 733–746. 10.1038/s41592-021-01116-4 33972782

[B55] Piomelli D. (2003). The molecular logic of endocannabinoid signalling. Nat. Rev. Neurosci. 4, 873–884. 10.1038/nrn1247 14595399

[B56] Quigley J. A. Logsdon M. K. Turner C. A. Gonzalez I. L. Leonardo N. B. Becker J. B. (2021). Sex differences in vulnerability to addiction. Neuropharmacology 187, 108491. 10.1016/j.neuropharm.2021.108491 33567305 PMC7979496

[B57] Quinones M. P. Kaddurah-Daouk R. (2009). Metabolomics tools for identifying biomarkers for neuropsychiatric diseases. Neurobiol. Dis. 35, 165–176. 10.1016/j.nbd.2009.02.019 19303440

[B58] Saigusa T. Aono Y. Mizoguchi N. Iwakami T. Takada K. Oi Y. (2008). Role of GABA B receptors in the endomorphin-1-but not endomorphin-2-induced dopamine efflux in the nucleus accumbens of freely moving rats. Eur. J. Pharmacol. 581, 276–282. 10.1016/j.ejphar.2007.12.008 18206140

[B59] Schrimpe-Rutledge A. C. Codreanu S. G. Sherrod S. D. McLean J. A. (2016). Untargeted metabolomics strategies-challenges and emerging directions. J. Am. Soc. Mass Spectrom. 27, 1897–1905. 10.1007/s13361-016-1469-y 27624161 PMC5110944

[B60] Shafik A. M. Allen E. G. Jin P. (2022). Epitranscriptomic dynamics in brain development and disease. Mol. Psychiatry 27, 3633–3646. 10.1038/s41380-022-01570-2 35474104 PMC9596619

[B61] Singh M. E. Verty A. N. McGregor I. S. Mallet P. E. (2004). A cannabinoid receptor antagonist attenuates conditioned place preference but not behavioural sensitization to morphine. Brain Res. 1026, 244–253. 10.1016/j.brainres.2004.08.027 15488486

[B62] Solinas M. Panlilio L. V. Antoniou K. Pappas L. A. Goldberg S. R. (2003). The cannabinoid CB1 antagonist N-piperidinyl-5-(4-chlorophenyl)-1-(2,4-dichlorophenyl) -4-methylpyrazole-3-carboxamide (SR-141716A) differentially alters the reinforcing effects of heroin under continuous reinforcement, fixed ratio, and progressive ratio schedules of drug self-administration in rats. J. Pharmacol. Exp. Ther. 306, 93–102. 10.1124/jpet.102.047928 12660305

[B63] Solinas M. Panlilio L. V. Tanda G. Makriyannis A. Matthews S. A. Goldberg S. R. (2005). Cannabinoid agonists but not inhibitors of endogenous cannabinoid transport or metabolism enhance the reinforcing efficacy of heroin in rats. Neuropsychopharmacology 30, 2046–2057. 10.1038/sj.npp.1300754 15870833

[B64] Song W. Wu C. F. Liu P. Xiang R. W. Wang F. Dong Y. X. (2013). Characterization of basal and morphine-induced uridine release in the striatum: an *in vivo* microdialysis study in mice. Neurochem. Res. 38, 153–161. 10.1007/s11064-012-0903-1 23070470

[B65] Spanagel R. (2017). Animal models of addiction. Dialogues Clin. Neurosci. 19, 247–258. 10.31887/DCNS.2017.19.3/rspanagel 29302222 PMC5741108

[B66] Strang J. Volkow N. D. Degenhardt L. Hickman M. Johnson K. Koob G. F. (2020). Opioid use disorder. Nat. Rev. Dis. Prim. 6, 3. 10.1038/s41572-019-0137-5 31919349

[B67] Sustkova-Fiserova M. Charalambous C. Havlickova T. Lapka M. Jerabek P. Puskina N. (2017). Alterations in rat accumbens endocannabinoid and GABA content during fentanyl treatment: the role of ghrelin. Int. J. Mol. Sci. 18. 10.3390/ijms18112486 29165386 PMC5713452

[B68] United Nations Office on Drugs and Crime (UNODC) (2023). World drug report 2023. Vienna: United Nations.

[B69] Viant M. R. Kurland I. J. Jones M. R. Dunn W. B. (2017). How close are we to complete annotation of metabolomes? Curr. Opin. Chem. Biol. 36, 64–69. 10.1016/j.cbpa.2017.01.001 28113135 PMC5337156

[B70] Vigano D. Valenti M. Cascio M. G. Di Marzo V. Parolaro D. Rubino T. (2004). Changes in endocannabinoid levels in a rat model of behavioural sensitization to morphine. Eur. J. Neurosci. 20, 1849–1857. 10.1111/j.1460-9568.2004.03645.x 15380006

[B71] Vigano D. Rubino T. Parolaro D. (2005). Molecular and cellular basis of cannabinoid and opioid interactions. Pharmacol. Biochem. Behav. 81, 360–368. 10.1016/j.pbb.2005.01.021 15927245

[B72] Volkow N. D. (2021). The epidemic of fentanyl misuse and overdoses: challenges and strategies. World Psychiatry 20, 195–196. 10.1002/wps.20846 34002497 PMC8129846

[B73] Volkow N. D. Wise R. A. Baler R. (2017). The dopamine motive system: implications for drug and food addiction. Nat. Rev. Neurosci. 18, 741–752. 10.1038/nrn.2017.130 29142296

[B74] Welsch L. Bailly J. Darcq E. Kieffer B. L. (2020). The negative affect of protracted opioid abstinence: progress and perspectives from rodent models. Biol. Psychiatry 87, 54–63. 10.1016/j.biopsych.2019.07.027 31521334 PMC6898775

[B75] Wise R. A. Robble M. A. (2020). Dopamine and addiction. Annu. Rev. Psychol. 71, 79–106. 10.1146/annurev-psych-010418-103337 31905114

[B76] Wu Y. Tao Y. Liang L. Wang Y. Xu G. Qu H. (2009). Metabonomic profile of rats with acute liver rejection. OMICS 13, 81–91. 10.1089/omi.2008.0061 19196102

[B77] Wu M. Sahbaie P. Zheng M. Lobato R. Boison D. Clark J. D. (2013). Opiate-induced changes in brain adenosine levels and narcotic drug responses. Neuroscience 228, 235–242. 10.1016/j.neuroscience.2012.10.031 23098802 PMC3525713

[B78] Xu W. He Y. Zhang J. Li H. Wan X. Li M. (2021). Simvastatin blocks reinstatement of cocaine-induced conditioned place preference in Male mice with brain lipidome remodeling. Neurosci. Bull. 37, 1683–1702. 10.1007/s12264-021-00771-z 34491535 PMC8643381

[B79] Yang Y. D. Zhang J. Z. Sun C. Yu H. M. Li Q. Hong M. (2006). Heroin affects purine nucleotides catabolism in rats *in vivo* . Life Sci. 78, 1413–1418. 10.1016/j.lfs.2005.07.014 16223513

[B80] Yao J. K. Dougherty G. G. Reddy R. D. Matson W. R. Kaddurah-Daouk R. Keshavan M. S. (2013). Associations between purine metabolites and monoamine neurotransmitters in first-episode psychosis. Front. Cell Neurosci. 7, 90. 10.3389/fncel.2013.00090 23781173 PMC3678099

[B81] Yu Q. He Z. Zubkov D. Huang S. Kurochkin I. Yang X. (2020). Lipidome alterations in human prefrontal cortex during development, aging, and cognitive disorders. Mol. Psychiatry 25, 2952–2969. 10.1038/s41380-018-0200-8 30089790 PMC7577858

[B82] Zaitsu K. Miyawaki I. Bando K. Horie H. Shima N. Katagi M. (2014). Metabolic profiling of urine and blood plasma in rat models of drug addiction on the basis of morphine, methamphetamine, and cocaine-induced conditioned place preference. Anal. Bioanal. Chem. 406, 1339–1354. 10.1007/s00216-013-7234-1 23912828

[B83] Zaitsu K. Hayashi Y. Kusano M. Tsuchihashi H. Ishii A. (2016). Application of metabolomics to toxicology of drugs of abuse: a mini review of metabolomics approach to acute and chronic toxicity studies. Drug Metab. Pharmacokinet. 31 (1), 21–26. 10.1016/j.dmpk.2015.10.002 26613805

[B84] Zelfand E. (2020). Vitamin C, pain and opioid use disorder. Integr. Med. (Encinitas) 19, 18–29. 33132774 PMC7572147

